# Correlations Between the Development of Social Anxiety and Individuals With Autism Spectrum Disorder: A Systematic Review

**DOI:** 10.7759/cureus.44841

**Published:** 2023-09-07

**Authors:** Jamal Montaser, Lotanna Umeano, Hari Priya Pujari, Syed Muhammad Zain Nasiri, Anusha Parisapogu, Anuj Shah, Safeera Khan

**Affiliations:** 1 Psychiatry, California Institute of Behavioral Neurosciences and Psychology, Fairfield, USA; 2 Internal Medicine, California Institute of Behavioral Neurosciences and Psychology, Fairfield, USA; 3 Diagnostic Radiology, California Institute of Behavioral Neurosciences and Psychology, Fairfield, USA

**Keywords:** autism, autism spectrum disorder, comorbid, social anxiety, social competence, social phobia

## Abstract

It is well established that people with autism spectrum disorder (ASD) have significantly higher rates of social anxiety, given that most autistic individuals experience socio-communication impairments, a deficit in social competence, and their experience in social engagement situations often leads to discomfort in social settings. Literature also finds that individuals on the spectrum are often at a higher risk of developing social anxiety, which is often misinterpreted as social anxiety disorder (SAD) leading to delays in the clinical diagnosis of ASD. Hence, an improved understanding of specific factors that put ASD individuals at risk of developing social anxiety will aid research to differentiate between social anxiety among individuals with ASD compared to non-ASD individuals facing social anxiety in general. This systematic review study focuses on empirical literature that provides evidence for reasons contributing to social anxiety among individuals with ASD. Following the systematic review methodology, the study evaluates 10 research papers. The results revealed several correlations that can be useful in helping explain why individuals with ASD are at a higher risk of developing SAD. Individuals with ASD often suffer severe social anxiety because they struggle to understand social cues, maintain eye contact, interpret non-verbal cues like facial expressions or body language, or participate in reciprocal conversation. Other cognitive factors include a preference toward predictable situations, intolerance for uncertainty, and a tendency toward rigid thinking patterns. Unpredictability in social settings often heightens anxiety levels in ASD individuals, making them avoid such situations. Other risk factors include emotional recognition impairments and reduced social competence. These findings serve as a guide to developing better intervention strategies to help individuals with ASD to overcome social anxiety.

## Introduction and background

Autism spectrum disorder (ASD) is a neurodevelopmental disorder characterized by difficulties in social communication and interaction and restricted and repetitive behavioral patterns. People with autism often have challenges in social interaction, such as difficulties understanding and expressing emotions, maintaining eye contact, and engaging in reciprocal conversations. ASD has been diagnosed in about one in 36 children [1]. It is widely accepted that many children and adults with ASD struggle with social anxiety disorder (SAD). SAD is a mental health condition characterized by an intense fear of social situations and constant concern about being judged, embarrassed, or humiliated by others. People with social anxiety might suffer from severe self-consciousness and constantly fear being negatively evaluated or scrutinized by others. Prevalence for social anxiety in autistic individuals has been estimated to be as high as 50%, significantly higher than the estimates of 7-13% for the non-ASD population [2].

Most children with ASD face frequent rejections in childhood, making them quiet and avoiding and withdrawing from engaging socially [3]. However, many of these characteristics are similar to those found in people with SAD, which has primarily confused the image of individuals with ASD leading many clinicians to conclude a false-SAD diagnosis and delays in establishing that patients are suffering from ASD. This is also because social anxiety is a common social phobia highly prevalent among the non-ASD population, particularly adolescents, with various long-term consequences [4].

Scholars studying non-ASD individuals with SAD and ASD indicate that the differentiating factor between the standard SAD and ASD-prone social anxiety is mostly covered in "why" the individuals may be experiencing anxiety. Theoretical frameworks in psychology have shown that social anxiety may occur due to adverse social experiences in childhood, perception of threat associated with social situations, lack of self-belief, impairment in attention and emotional processing, inhabitant temperament, and other coping behaviors such as mental rehearsal, avoidance, etc. [2]. Other reasons for misinterpretation of ASD diagnosis among individuals may be because many people on the spectrum of autism possess high cognitive skills and have had years of enhancing cognitive learning processes to compensate for other social and engagement-related impairments [5]. Therefore, the current review explores the present literature for correlations and reasons for social anxiety among individuals with ASD and whether these reasons differ for those suffering from SAD. More effective interventions may result from an increased awareness of the particular components that contribute to the development of social anxiety in ASD.

## Review

Methods

This systemic review used the Preferred Reporting Items for Systemic Reviews and Meta-Analysis (PRISMA) 2020 guidelines [6].

Search Sources and Strategy

We searched for relevant articles through PubMed, PubMed Central, Medline, and ScienceDirect. We used various combinations of social anxiety, social phobia, and autism spectrum disorder to search all databases. However, with PubMed, along with these keywords, the following strategy was created and utilized to look for relevant articles in the MeSH database: ("autism spectrum disorder/complications"[Mesh]) AND (( "phobia, social/complications"[Mesh] OR "phobia, social/etiology"[Mesh] )). Table 1 shows the databases used and the identified numbers of papers for each database.

**Table 1:** Keywords/strategy used and the number of identified papers

Search strategy	Database used	Number of papers identified
“Autism spectrum disorder [all]” AND “comorbid social anxiety [all]”	PubMed (advanced field search)	725
("Autism spectrum disorder/complications"[Mesh]) AND (( "phobia, social/complications"[Mesh] OR "phobia, social/etiology"[Mesh] ))	PubMed (MeSH) database	4
"Autism spectrum disorder" and causes of "social anxiety"	Science Direct	1,312
Total number of research papers identified		2,041
Number of articles after removing duplicates		2,037

Inclusion and Exclusion Criteria

We selected articles that were written and published in the English language and that focused on either adults or children. Only articles focusing solely on social anxiety were included.

Articles were excluded if a free full-text version of the paper could not be retrieved. Articles focusing on other mental health disorders in autism patients were also excluded. Grey literature was not included.

Selection Process

We removed any articles that did not have full text available. However, before doing that, all 2,037 articles were screened solely by title to make sure none of the articles without full text available are of notable importance to the study. This decreased significantly any shortcomings of the study. We then transferred the shortlisted articles to Endnote (Clarivate Plc, London, United Kingdom) and removed any duplicate papers. Each article was screened through titles and abstracts. The shortlisted articles were further evaluated by evaluating the full text, and only relevant articles were assessed. Only articles that met the criteria for inclusion and exclusion were selected for shortlisting.

Quality Assessment of the Studies

The appropriate quality appraisal tools were used to evaluate the shortlisted articles. Non-randomized clinical trials and observational studies were assessed for quality using the Newcastle-Ottawa tool, while narrative reviews were evaluated using the Scale for the Assessment of Narrative Review Articles (SANRA). Only studies that satisfied the quality appraisal were included in the systematic review.

Data Collection Process

After the articles were finalized for the systematic review and extracted, the primary outcomes were assessed along with other necessary information. We tabulated data about the study design, sample size, participant demographics in clinical and comparator groups, outcome measures employed, and study results.

Results

Study Identification and Selection

**Figure 1:**
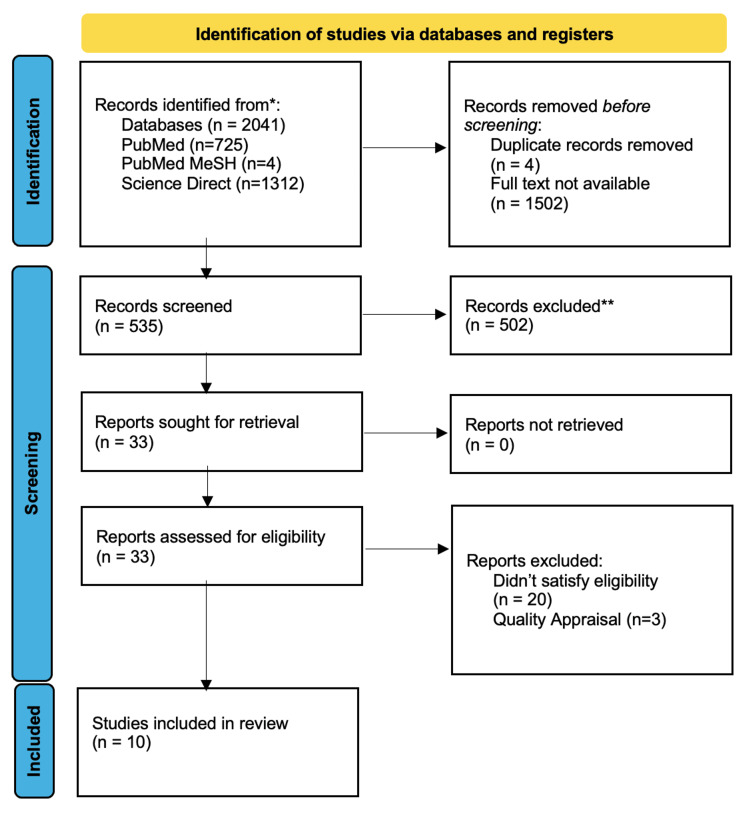
PRISMA flowchart showing the process of article selection

We identified a total of 2,041 relevant articles using all databases. Four duplicate articles and 1,502 articles with no full text available were removed before screening them in detail. After screening these articles by reviewing titles and abstracts, 33 were shortlisted. The shortlisted full-text articles were assessed for eligibility and quality, and 10 were finalized for review. The selection process of the studies is shown in Figure 1 in the PRISMA flowchart.

The articles were assessed for eligibility using the relevant quality appraisal tools. Tables 2-3 show the results of the quality appraisal.

**Table 2:** A quality appraisal using SANRA

Study	Importance to readers	Aims of the narrative reviews	Description of the literature search	Referencing	Scientific reasoning	Appropriate presentation of data	Total score
South and Rodgers 2017 [7]	2	2	0	2	2	2	10
White et al. 2014 [8]	2	2	0	2	1	2	9

**Table 3:** A quality appraisal using the Newcastle-Ottawa tool

Study	Selection	Comparability	Outcome
Espelöer et al. 2020 [9]	****	*	**
Meyer et al. 2006 [10]	****	**	**
Jobe and White, 2007 [11]	***	**	**
Bejerot et al. 2014 [12]	****	**	**
Stark et al. 2023 [13]	****	**	*
White et al. 2015 [14]	****	**	**
Briot et al. 2020 [15]	****	*	**
Corden et al. 2008 [16]	****	**	**

Outcomes Measured

The primary outcomes from the finalized research papers were the factors that tell us why individuals with ASD are at a higher risk of developing comorbid SAD, such as low social competence, impaired emotion recognition, and other reasons discussed (Table 4).

**Table 4:** Summary of variables identified in selected studies ASD: autism spectrum disorder, SAD: social anxiety disorder, SASKO: social anxiety-social competence deficit scale, IU: intolerance for uncertainty scale

Author(s)	Country	Study characteristics	Population	Results
South and Rodgers 2017 [7]	United States and United Kingdom	The review-based study focuses on three aspects of ASD; atypical sensory function and anxiety, alexithymia and Mindfulness in ASD, and intolerance of uncertainty in ASD to suggest a model for future studies.	Youth and adults	The study suggests that the intolerance of uncertainty is an intermediate factor in the development of anxiety in social settings, which makes it hard for ASD individuals to live with ambiguity and find it challenging to identify whether, under each social circumstance, the same rules will be followed. This is also because ASD individuals are cognitively and behaviourally rigid, which relates to having repetitive and restricted behaviors. Other reasons that contribute to social anxiety include a lack of self-emotional awareness in complex situations.
White et al. 2014 [8]	United States	The study reviews to understand anxiety among people with ASD.	Children, adolescents, and adults	It is suggested that for anxiety in ASD, emotional regulation impairments are a risk factor. The ubiquitous link between emotional regulation impairments and ASD that stems from physiological, socio-cognitive, and neural mechanisms answers why individuals with ASD are at a higher risk of developing comorbid SAD.
Espelöer et al. 2020 [9]	United Kingdom	The study is based on a quasi-experiment design for ASD individuals (n=25; mean age: 38.8), non-clinical individuals (n=23, mean age: 44), and individuals in the reference group with SAD (n=68, mean age: 37). The authors measure differences in SASKO and IU scores among the groups.	18 to 65 years old individuals	The study finds that individuals in the ASD group scored significantly higher on social anxiety values of speaking and rejection than those in the SAD group. Furthermore, a deficiency in social competence, which includes deficits in interaction and difficulty in processing social information, is another factor that contributes to social anxiety among autistic individuals.
Meyer et al. 2006 [10]	United States and United Kingdom	The authors experiment on individuals with Asperger's syndrome (n=31) and typical development children (n=33) by examining children’s responses to hypothetical social vignettes, self-reported social difficulties, and psychological functioning measures. Interviews were also conducted with parents to gain detailed information on the clinical diagnosis of their children.	Children	The study found a systematic correlation between self-reported social anxiety by children and their parents' reports of impaired social competence. The study suggests that social information and attribution processing may be important factors to consider in understanding the emotional and behavioral difficulties experienced by children with Asperger's syndrome.
Jobe and White 2007 [11]	United States	The quantitative study uses a sample of 97 undergraduate individuals (mean age: 19.4) to measure characteristics of dating, friendship history, feelings of loneliness, and social motivation experienced by autistic individuals. The data was collected on a self-reporting basis through a survey-based questionnaire. The study measured a broader autism phenotype, which refers to a set of subclinical personality traits and symptoms associated with ASD. The study used the Autism-Spectrum Quotient to measure the broader autism phenotype in the participants.	College students	The study found that individuals with a stronger phenotype had romantic relationships of longer duration, in comparison to those with fewer ASD characteristics. The study also found that the total autism-spectrum quotient score was a valid predictor of loneliness, and the contribution made by the length of the current best friendship approached significance. However, there was a significant correlation between respondents with high autistic spectrum scores and the need for long-term romantic relationships. Preference for sameness and resistance to change are hallmark characteristics of ASD. There is a possibility that this plays a role in the tendency for individuals with stronger ASD phenotypes to maintain long-term romantic relationships.​​​​​​
Bejerot et al. 2014 [12]	Sweden	Researchers employed a comparative survey design, with three groups: individuals with ASD (n=50, mean age: 30 years), individuals with SAD (n=100, mean age: 34.6 years), and a comparative group including non-ASD (n=53, mean age: 32.3 years) people.	Adults	Even though individuals with ASD and SAD showed a high level of social anxiety, the reasons were different. The SAD group perceived themselves as being socially incompetent even though they acknowledged adequate social skills; however, people with ASD are more socially awkward and have poor social skills. The study also found that some ASD individuals often have less social anxiety due to their poor insight into how others perceive them.
Stark et al. 2023 [13]	United States	Based on a secondary data analysis, the research evaluates a sample of 194 participants with and without psychiatric disorders. It includes the measures related to autism spectrum symptoms, social anxiety symptoms, social competence, intelligence quotient, and socioeconomic status.	Children (8-13 years old)	According to the results, reduced social competence is a risk factor for social anxiety development among children with ASD, and the relationship between social anxiety and ASD is not solely attributable to measurement/item overlap.
White et al. 2015 [14]	United States	The study includes 15 participants with ASD and 18 (gender-matched) without ASD. The measures include brief fear of negative evaluation, social worries, social responsiveness, social communication, and gaze patterns.	Adolescents (12-17 years)	The study finds that adolescents with ASD, who are more cognitively able, are more like to have social anxiety. Negative evaluation’s self-reported fear among adolescents with ASD who are cognitively unimpaired explained a higher duration of gaze to social threat cues.
Briot et al. 2020 [15]	France	The research recruited 79 participants with ASD and 28 matched control participants. It screens anxiety disorders, psychiatric comorbidities, and depression using standard tools.	Children and adolescents; mean age = 11.51 years	A high risk for anxiety disorders is observed among individuals with ASD. Moreover, high social communication and motivation impairment levels indicate ASD and social anxiety (comorbid disorders).
Corden et al. 2008 [16]	United Kingdom	The study includes 21 individuals with a clinical diagnosis of Asperger’s syndrome. It also recruits 21 gender-matched healthy controls. The subjects completed various tests and tasks, including facial affect recognition, eye tracking, and a self-report measure of social anxiety.	Adults (M = 33.8 years) and controls (M = 32.1 years)	The study finds that, at recognizing fearful and sad facial expressions, the participants with Asperger’s syndrome performed significantly worse than the control group without Asperger's syndrome. However, it is also observed that perceptual or cognitive ability differences do not explain the sadness and fear recognition score’s variability. The participants with Asperger’s syndrome made significantly fewer fixations to the eyes, which affected their fear recognition ability. Findings conclude that the data supports an "amygdala hyperactivity" model that may explain the suggested lack of attendance to social stimuli in individuals with Asperger's syndrome.

Discussion

Individuals with ASD are often observed to experience anxiety in social settings. The systematic review's findings confirm that social anxiety among ASD individuals significantly differs from the typical SAD that non-ASD individuals go through in terms of social awkwardness, experiences, and behaviors.

Relating the findings to the research question, the studies by South and Rodgers and Espelöer et al. commonly highlighted that a high prevalence of intolerance to uncertainty (IU) among individuals with ASD often puts them at risk of social anxiety [7,9]. Previous literature refers to IU as a personality characteristic where individuals have difficulty perceiving ambiguity and exhibit discomfort with uncertainty [17]. Even though IU is not at the core of ASD, many autistic individuals may experience IU because they have heightened sensory sensitivities, which can make uncertain or unpredictable sensory stimuli overwhelming [18]. This often leads them to prefer routine and predictable situations where they can follow standard rules. Individuals with ASD experience discomfort if their environment changes or any unexpected change occurs in their daily setting, making them more anxious around new people and complex social situations. Hence, social interactions for ASD-prone individuals can be challenging as they cannot understand social cues and norms in different settings. Therefore, uncertain social situations, such as unfamiliar social contexts or ambiguous social signals, can increase anxiety, contributing to difficulty for individuals with ASD to navigate social interactions.

The study presented by Espelöer et al. included the concept of intolerance of uncertainty and found that it represents an intermediate factor in the mechanism of anxiety development in adults with ASD. The researcher studied the interaction impact of IU on ASD and non-ASD individuals to determine social anxiety levels and concluded that increasing level of discomfort with ambiguity leads to a higher deficit in social competence, making individuals with ASD avoid social settings, which further limits their opportunities to practice and acquire social skills and improve communication capabilities [19]. Similarly, the study of South and Rodgers also proposed a framework of anxiety symptoms among ASD individuals where IU plays a critical mediating role [7]. Concerning the suggested framework, it is concluded that intolerance of uncertainty among ASD individuals comes from medial prefrontal cortex (mPFC) dysfunction, which increases social disabilities. The authors discussed that mPFC leads to "difficulties with uncertainty at the primitive level," which appear "to be related to difficulties in flexible emotional response" [7]. This is also because people with ASD often cannot regulate their present experience and social cues with information from past experiences, ultimately overwhelming them with new information.

The inability to understand social cues comes from another common factor highlighted through the systematic review: the "lack of self-awareness" in emotions, which further contributes to social anxiety among ASD individuals. This factor has been discussed in detail by South and Rogers, White et al., and Meyer et al. [7,8,10]. White et al. explained that emotional regulation (ER) impairment also puts individuals with ASD at a higher risk of developing comorbid SAD [8]. Previous research suggests that, in social situations, ER impacts an individual’s ability to cope and manage emotions. This could be explained by reduced social cognition performance that negatively influences the ability to recognize and interpret social cues, leading to social anxiety in ASD [19]. It is observed that ER impairments may result in different behavioral issues among individuals with ASD, including anxiety [20]. However, White et al. emphasized certain mediators (physiological, socio-cognitive, and neural mechanisms) and moderators (social motivation, attention/bias, avoidance, etc.) of ER impairment in ASD, resulting in anxiety [8].

In the same context, Meyer et al. studied children with Asperger's syndrome who are vulnerable to comorbid psychiatric symptoms of anxiety and depression [10]. The authors compared two groups, one with Asperger's syndrome and the other with typical development, and examined their self-reported and parents-reported extent of social functioning/awareness, social information, attribution processing, and measures of social-cognitive ability. The study's results discussed that children with Asperger's syndrome reported high levels of anxiety and depression and a lack of satisfaction and competence in interpersonal relationships. Even though their self-reports were unrelated to their parents' reports, the children exhibited experiences of emotional and social challenges. Similar to the results of South and Rogers on information processing, Meyer et al. also stated that children’s social and emotional difficulties are related to their lack of social information processing tendencies. The authors also pointed out that children in the Asperger's syndrome group presented a lack of capability to understand social scenarios and generated adaptive repeated responses more than children in the comparison group. This shows that the children at risk of ASD cannot generate a variety of responses that would be suitable in different social settings.

These factors relate to the need for "sameness and resistance to change," which was explained in detail by Jobe and White, who quantitatively studied feelings of loneliness, attention to detail, switching, communication, imagination, length of friendships, and dating relationships among respondents who completed a self-reported autism spectrum quotient (AQ) scale [10]. The researchers determined that respondents who scored high on the AQ scale have a significantly lower length of best friendships, having a short duration of friendships. It should also be noted that social motivation was not correlated to the AQ score, and the need to make new friends or keep old friends was negatively correlated with the AQ score. On the contrary, the results observed a positive correlation between the AQ score and the length of romantic relationships. The authors justified these results by explaining that individuals with stronger ASD phenotypes do not necessarily lack social motivation but do not have the required social skills and understanding to sustain long-term friendships. The findings of Bejerot et al. came under a similar strand of research where the authors studied three different groups of adults, one with ASD, one with SAD, and another non-ASD comparison group [8]. The results showed that people with SAD acknowledge their lack of appropriate social skills and social incompetency; however, adults with ASD are socially awkward and lack that insight. Bejerot et al. explained that poor insight or awareness about being socially awkward could also protect them from social anxiety.

This research further talks about reduced social competence as a risk factor for social anxiety in children with ASD based on the study of Stark et al. [13]. The characteristic of reduced social competence is common in both ASD and social anxiety. It is considered a risk factor since it creates difficulties in establishing essential social skills that lead to successful social interactions [13]. The symptoms of social anxiety in ASD could be attributed to autism-specific social skills deficits. It means that social competence deficits are related in the case of ASD, while the symptoms of social anxiety are a consequence of social competence skills disturbance [9].

White et al. also discussed similar concepts and found that more cognitively able adolescents with ASD are more likely to have social anxiety [14]. It could be attributed to a greater level of awareness of social difficulties. In social situations, this could result in greater self-consciousness and social anxiety [13]. High social communication and social motivation impairment levels are also among the risk factors for anxiety, according to Briot et al. [15]. For successful social interactions, these factors (similar to those discussed earlier) are linked with difficulties in establishing essential social skills [21], resulting in higher social anxiety among individuals with ASD. It is also observed that anxiety symptoms negatively impact social deficits in ASD [22], and the consequences could be social avoidance, impaired social communication, and increased social sensitivity. It suggested that the link between social deficits and anxiety in ASD is bidirectional, i.e., social deficits lead to anxiety, while anxiety symptoms could also exacerbate social deficits.

According to Corden et al., reduced fixation of the eyes and poor fear recognition are linked with higher social anxiety levels in Asperger’s syndrome individuals, where fewer fixations to the eyes influence their fear recognition ability [16]. Previous research confirmed that high social anxiety is associated with correctly identifying fearful expressions in adolescents [23]. Moreover, reduced fixation of the eyes in ASD is a common trait. It is observed that early avoidance of the eyes is associated with social anxiety among students with high ASD [24]. It suggested the reduced fixation of the eyes and poor fear recognition ability influence social anxiety among individuals with ASD since these factors impact the cues and information processing ability necessary for social interaction. Based on the above discussion of the current systematic review study, while supporting evidence from other literature, it is observed that there are several correlations that can be made for the development of social anxiety in individuals with ASD.

Previous literature recognized the link between ASD and SAD; however, future research could further deepen our understanding of the given link. In this regard, more longitudinal studies following individuals with ASD could help investigate the progression and development of symptoms of social anxiety over time. This would help identify the critical periods for interventions or relevant therapies. Moreover, there is a need to explore further the processes related to social cognition, which contribute to social anxiety development, such as understanding social attribution biases and emotion recognition. Future research may also investigate environmental factors’ role, such as school environment, family functioning, and early social experiences in the social anxiety developing in the case of ASD. This could also help in suggesting support strategies and interventions. It is also suggested to examine individual differences, i.e., why social anxiety is developed in some individuals with ASD and not in all; this could be done by exploring the adaptive skills, sensory sensitivities, cognitive profiles, and comorbidities to determine higher-risk subgroups. Moreover, future research may examine potential gender and other demographic differences in the co-occurrence of the two disorders, which could help inform tailored intervention approaches. These recommendations can help gain a detailed understanding of the relationship between ASD and social anxiety and the underlying risk factors, protective factors, and mechanisms linked to the co-occurrence of the two disorders.

Limitations

Fewer studies focused on cognitive disabilities such as mPFC and reduced fixation of the eyes, which may have been more significant in causing or maintaining social anxiety among individuals than other reasons. Nevertheless, studies that were reviewed did not compare which of these symptoms of ASD may result in greater levels or lower levels of anxiety, which may make up for one of the limitations of the current research. The small sample of papers reviewed was also a limitation of the study. Apart from that, the research considered studies in the English language, omitting many non-English language publications which may have added further useful information.

## Conclusions

Narrative synthesizing of the literature indicated that social anxiety in individuals with ASD significantly differs from SAD in non-ASD adults or children. This is mainly because the factors that induce anxiety among ASD individuals are different. Even though the core social communication deficits in ASD are not caused by social anxiety, per se, several correlations can be made that may explain why individuals with ASD are at a higher risk of developing social anxiety. The review revealed that social anxiety in ASD individuals is associated with emotional regulation impairment, specific cognitive deficits, intolerance for uncertainty, reduced social motivation, lack of information processing, the rigidity of repeated behaviors, and reduced fixation of the eyes leading to poor fear recognition in social settings. Based on the literature, many of these symptoms might contribute to or perpetuate social anxiety. Understanding these factors can lead to prevention, early detection, and more effective interventions in individuals with ASD.
