# Correction: Correlations Between the Development of Social Anxiety and Individuals With Autism Spectrum Disorder: A Systematic Review

**DOI:** 10.7759/cureus.c311

**Published:** 2025-09-23

**Authors:** Jamal Montaser, Lotanna Umeano, Hari Priya Pujari, Syed Muhammad Zain Nasiri, Anusha Parisapogu, Anuj Shah, Safeera Khan

**Affiliations:** 1 Psychiatry, California Institute of Behavioral Neurosciences and Psychology, Fairfield, USA; 2 Internal Medicine, California Institute of Behavioral Neurosciences and Psychology, Fairfield, USA; 3 Diagnostic Radiology, California Institute of Behavioral Neurosciences and Psychology, Fairfield, USA

This article has been corrected to fix the following typo in the PRISMA diagram: Reports excluded due to not satisfying eligibility criteria corrected from 23 to 20.

